# Prevalence and correlates of non-medical stimulants and related drug use in a sample of South African undergraduate medical students

**DOI:** 10.4102/sajpsychiatry.v22i1.795

**Published:** 2016-06-24

**Authors:** Mari Retief, Chris Verster

**Affiliations:** 1 Faculty of Medicine and Health Sciences, Department of Psychiatry, Stellenbosch University, South Africa

## Abstract

**Background:**

The non-medical use of prescription psychostimulants or cognitive-enhancing substances among healthy college students is a growing concern. This use appears to be particularly high among medical students. To our knowledge, no literature is available on the non-medical use of stimulants among South African medical students.

**Objective:**

To determine the prevalence and correlates of non-medical stimulant use as well as subjective opinion on peer numbers using stimulants and university attitude towards stimulant use among a sample of South African undergraduate medical students.

**Methods:**

A descriptive observational study was conducted by means of a self-report questionnaire. Second- and fourth-year medical students (*n* = 252) completed the questionnaire.

**Results:**

Of the sample, 44 (18%) reported a lifetime use of stimulants for non-medical purposes and 33 (85%) of this group reported use within the past year. A total of six (2%) students reported a diagnosis of Attention Deficit/Hyperactivity Disorder (ADHD). In the group without a diagnosis of ADHD, non-medical stimulant use was associated with the year of study (*p* = 0.03) and illicit substance use (*p* = 0.01). Most of the students in this group (31, 32%) reported using stimulants to improve concentration.

**Conclusion:**

Non-medical use of stimulants to improve concentration and academic performance is prevalent among the South African medical students sampled in this study. Further research at other institutions and under non-medical students would be helpful to assess the scope of this phenomenon.

## Introduction

The non-medical use of prescription psychostimulants or cognitive-enhancing substances among healthy college students is a growing concern. Stimulant drugs are generally prescribed for the treatment of Attention Deficit/Hyperactivity Disorder (ADHD), narcolepsy and some cases of depression.^1^ Yet, reports show that 5% – 35% of college students use prescription psychostimulants for non-medical purposes.^2^ These drugs are normally prescribed to increase motivation, mood, energy and wakefulness, and include methylphenidate, dextroamphetamine, pemoline and modafinil.^1^

Abuse of stimulant drugs can lead to psychological and physiological tolerance and dependence. Common side effects include gastrointestinal discomfort, anxiety, irritability, insomnia, tachycardia, cardiac arrhythmias and dysphoria. Less common adverse effects include induction of movement disorders, Tourette’s disorder and other tics. In addition, high doses of sympathomimetics (which might be the case if stimulants are used non-medically or recreationally) can lead to dry mouth, bruxism, formication, emotional liability, psychosis and seizures.^1^ Besides the above concerns, there are multiple ethical considerations involved. In the broader context, these include justifying treatment to healthy or subsyndromal individuals (referring to individuals displaying some symptoms of an illness but not severe enough for diagnosis as a clinically recognised syndrome), as well as offering pharmacological neuro-enhancement for doctors working long shifts and other occupations requiring prolonged attention such as long-distance truck drivers or pilots. Lastly, the possible need for the development of protocols on the use of psychostimulants by academic institutions is another area of possible ethical debate.^3,4^

In 2001, a nationwide survey in the United States on the non-medical use of stimulants among a group of 10 000 undergraduate university students revealed a lifetime prevalence of 6.9%, a past year prevalence of 4.1% and a past month prevalence of 2.1%.^5^ This cohort comprised of randomly selected students attending various 4-year courses at 119 American colleges and universities. A cross-sectional assessment was done with the help of a 20-page survey. In 2005, Teter et al.^6^ reported 8.1% lifetime prevalence and a 5.4% past-year prevalence of illicit prescription stimulant use from an Internet survey of 9161 randomly sampled college students. These students were all from a single large public university and were invited via email to complete the Student Life Survey (SLS), developed by the Michigan Substance Abuse Research Centre. Furthermore, reports show that medical students appear to be at higher risk for stimulant use: in 2010, Tuttle et al. reported that 10.0% of medical students used stimulants during their lifetime.^7^ Another report showed that 20.0% of medical students used stimulants during their lifetime and 15.0% used during medical school.^8^ It should, however, be noted that 9.0% of participants in the aforementioned study were diagnosed with ADHD.^8^ In 2013, Emanuel et al.^2^ conducted a multi-institutional census on 2732 medical students and reported a lifetime psychostimulant use of 18.0%, with 11.0% reporting use during medical school and 63.0% of those using stimulants non-medically. This was an online, anonymous, cross-sectional survey about cognitive enhancement drug use and associated factors to all enrolled students at four Chicago-area medical schools, one public and three private institutions, across class year one through to six.

These above-mentioned studies found that the reasons for non-medical use of stimulants are diverse, including coping with the pressure of an academic environment, improving school performance, staying awake to study or complete projects, as well as recreationally to achieve euphoria or lose weight.^2^ Other factors associated with higher rates of use included being male, white, members of fraternities and/or sororities and lower average grades.^5^ Non-medical use was also associated with concomitant high-risk activities, such as use of alcohol and illicit drugs as well as driving while intoxicated.^2,9^

To our knowledge, there is no formal information on non-medical stimulant use at South African colleges or universities. A 2011 report in the South African lay press stated that diversion (where a person with a prescription for stimulants will sell or give his and/or her medication to peers) and the non-medical use of stimulants is fairly common at our universities.^10^ With this in mind and the fact that the medical fraternity seems to be particularly vulnerable to stimulant use, the aim of this study was to determine the prevalence and correlates of non-medical sympathomimetic use as well as subjective opinion on peer numbers using these drugs and university’s attitude towards this use among a sample of undergraduate medical students at a South African university. Specific objectives included: (1) assessing the reasons for use, source of stimulants, effectiveness and medical, as well as psychiatric comorbidities; and (2) students’ perception on peer frequency of use and the attitude of the university towards stimulant use. Examples of stimulants included methylphenidate preparations, atomoxetine and modafinil, whereas cognitive abilities were defined as the ability to concentrate/think clearly/learn/remember.

## Methods

### Design and setting

This was a descriptive observational study conducted at the medical campus of a South African university.

### Participants

The sample consisted one class each of second- and fourth-year medical students. There were no exclusion criteria for this study and all students from these classes were included in the survey. We included both junior and more senior students to assess the association between stimulant use and age and/or year of study. These two classes were least involved in research according to the specific university regulations and therefore available for our study.

### Data collection

Data were analysed using Statistica version 11 of 2013. Age was the only continuous variable and was described using means and standard deviations. Furthermore, the analysis of age was stratified according to questions 7 and 18 of the questionnaire (referring to presence or absence of an ADHD diagnosis and non-medical use of sympathomimetics). All other variables were analysed descriptively by means of frequency distributions. Additionally, contingency tables were produced whereby the responses were analysed according to the responses obtained from questions 7 and 18. A histogram was used to graphically present age and bar charts for all other nominal variables. As age was not normally distributed, a non-parametric Mann–Whitney U test was used to compare whether the ages differed with respect to question 18. A comparison of two nominal variables was performed using a contingency table and Pearson’s chi-square test. In the event of small, expected, cell frequencies, a Fisher’s exact test was applied. A significance level of 5% was applied throughout.

### Ethical considerations

Ethical approval was granted by the Health Research Ethics Committee of Stellenbosch University, while lecturers and the appropriate university authorities also granted permission to conduct the study. Ethical standards as put forth by the Health Research Ethical Committee of Stellenbosch University were strictly adhered to. Each student signed a Participant Information Consent Form (PICF) and was handed an anonymous questionnaire in exchange for the signed PICF. Each questionnaire was identified with a research number only and remained completely confidential.

## Results

### Demographic data

A total of 252 questionnaires were completed. One questionnaire was excluded from the analysis due to contradictory information. The final sample hence amounted to 251 (142 second-year students and 109 fourth-year students). Participants had a mean age of 20.9 (s.d. 1.8, range 19–32), and 184 (73%) were women and 67 (27%) men. Those who lived in residential facilities amounted to 147 (56%), while 103 (41%) lived privately. Most students had a grade average between 50 and 70 (163, 64%).

### Prevalence of stimulant use

Of the students, 42 (17%) reported a lifetime use of sympathomimetics for non-medical purposes and 33 of this group (79%) reported use within the past year.

### Diagnosis of attention deficit/hyperactivity disorder

A total of 6 (2%) students reported a diagnosis of ADHD and 3 (50%) were male. Of the students with ADHD, 3 (50%) were diagnosed by a psychiatrist, 2 (33%) by a general practitioner and 1 (17%) by a Grade 6 teacher. Half of the students with ADHD were on treatment. All the students with ADHD were in their second year of study and resided in residential facilities. All of the students diagnosed with ADHD used alcohol in the month prior to completing the questionnaire and 1 student (17%) also reported use of illicit substances concomitantly with stimulant treatment. One student (17%) reported diversion behaviour.

### Reason for use, source of stimulants, effectiveness and comorbidities in the non-attention deficit/hyperactivity disorder group

The majority (31; 32%) reported using stimulants to improve concentration (Table 1). Of the students, 18 (44%) obtained stimulants free from a friend or classmate, 4 (9%) bought stimulants from a friend or classmate, 15 (37%) obtained stimulants from other sources (a shop, a pharmacy, via the Internet using Swiss Guard, over the counter, or ‘house shop’) and 1 (2%) did not respond. Some of the students (4, 9%) reported obtaining the stimulants from a family member with a diagnosis of ADHD. The majority of students found the stimulants to be moderately effective (30; 75%). Regarding comorbidities, 20 (9%) of the students were diagnosed with a psychiatric illness and 6 (3%) suffered from a cardiovascular ailment.

**TABLE 1:** Reasons for use of stimulants.†

Variable	*n* = 42	%
To improve concentration	31	32
To stay awake	23	24
To improve academic performance	18	18
For increased energy	18	18
To party/for recreational use	5	5
To lose weight	1	1
To counter effects of other drugs	0	0

† Students were allowed to choose multiple reasons; values given as percentage of total response.

### Correlates of stimulant use between users and non-users in the non-attention deficit/hyperactivity disorder group

There was a significant difference between users and non-users in terms of year of study (*p* = 0.03), with the majority of users in their second year of study (30; 72%). Further significant differences between the groups occurred with illicit substance use (*p* = 0.01), with the stimulant users using more illicit substances and for stimulant side effects (*p* = 0.002), with the group using stimulants being more informed about side effects of neurostimulants. There were however no significant differences between the groups in terms of age (*p* = 0.43), sex (*p* = 1.00), living arrangements (*p* = 1.00), grade average (*p* = 0.29) or alcohol use (*p* = 0.07) (Table 2).

**TABLE 2:** Correlates of non-medical stimulant use in undergraduate medical students.†

Variable	Stimulant use						*p*

	Yes		No				
	
	*n*	%	*n*	%			
**Age**					0.43
Mean	21.06	-	20.86	-	
s.d.	1.74	-	1.85	-	
Range	19–32	-	19–27	-	
**Gender**					1.00
Male	11	26	53	27	
Female	31	74	147	74	
**Year of study**					0.03
Second year	30	71	103	51	
Fourth-year	12	29	97	49	
**Grade average**					0.29
50% – 70%	31	73	126	63	
70% – 80%	2	22	62	32	
> 80%	9	5	4	2	
< 50%	0	0	5	3	
**Living arrangements**					1.00
Private	18	43	84	42	
Residential	24	57	115	58	
**Frequency of use**					1.00
Past year	33	85	0	0	0.31
Past month	4	10	0	0	
Past week	2	5	0	0	
Once	14	30	0	0	
> 5 times	12	30	1	0.5	
2–5 times	16	40	0	0	
**Perceived effectiveness**					0.44
Moderately effective	30	75	3	60	
Very effective	7	18	2	40	
Not effective	3	8	0	0	
**Source of stimulants**					0.20
Free from peer	18	44	0	0	
Bought from peer	4	9	0	0	
‘Faked ADHD’	0	0	0	0	
Family member	4	9	-	-	
Other	15	37	3	100	
**Cardiovascular illness**					0.29
Yes	2	5	4	2	
No	40	95	191	98	
**Psychiatric illness**					0.75
Yes	4	10	16	8	
No	37	90	179	92	
**Aware of stimulant side effects**					0.002
Yes	27	64	74	38	
No	15	36	121	62	
**Past month alcohol use**					0.07
Yes	24	57	105	54	
No	18	43	92	47	
**Past month substance use**					0.01
Yes	6	15	7	4	
No	34	85	187	96	

† Students without a diagnosis of ADHD, comparing stimulant users (‘yes’) and non-users (‘no’).

### Students’ opinion on the number of peers using stimulants

There was no significant difference (*p* = 0.32) between users and non-users in the non-ADHD group relating to opinion on peer frequency of sympathomimetic use. Most students in both user (25; 60%) and non-user (106; 54%) groups responded that only a few of their peers used these drugs (Table 3). Furthermore, in the non-user group, a large number of students (60; 30%) felt that none of their peers were using stimulants while in those with ADHD, 50% (3) felt that most of their peers were using stimulants.

**TABLE 3:** Students’ opinion on frequency of peer stimulant use.†

Variable	None		Only a few		Many		Most		Do not know	
				
	*n*	%	*n*	%	*n*	%	*n*	%	*n*	%
Students diagnosed with ADHD	0	0	3	50	3	50	0	0	0	0
**Students *not* diagnosed with ADHD**									
Using stimulants	7	17	25	60	9	22	0	0	1	2
Not using stimulants	60	30	106	54	25	13	1	0.5	6	3

† Students were asked: ‘How many of your fellow students use stimulant mediation to improve cognitive abilities?’.

### Students’ opinion on university attitude towards stimulant use

There was a significant (*p* = 0.00) difference between users and non-users in the non-ADHD group for opinion on the university’s attitude towards stimulant use. The non-users felt more strongly than the users that the university should prohibit stimulant use, whereas the users felt more strongly that the university’s attitude towards stimulant use should be neutral. The ADHD group was divided between ‘neutral’ (4; 66%) and ‘discouraging’ (2; 33%). Furthermore, there was a significant (*p* = 0.00) difference of opinion between the users and non-users in the non-ADHD group on whether the non-medical use of stimulants is academic ‘cheating’, with the majority of users responding ‘no’ (see Table 4).

**TABLE 4:** Students opinion on university’s attitude.†

Variable	Discourage		Prohibit use		Neutral		Encourage	
			
	*n*	%	*n*	%	*n*	%	*n*	%
Students diagnosed with ADHD	2	34	0	0	4	66	0	0
**Students *not* diagnosed with ADHD**							
Using stimulants	11	26	1	2	24	57	6	14
Not using stimulants	75	38	53	27	65	33	3	1

† Students were asked ‘What should the university’s attitude be towards the use of stimulants?’

## Discussion

This report presents to our knowledge the first findings on rates and correlates of non-medical use of stimulants and related drugs in South African undergraduate medical students. Our results showed that 17% of our sample used stimulants during their lifetime, with only 2% of the sample having a diagnosis of ADHD. The prevalence of use in our sample mirrors international figures obtained by Emanuel et al.^2^ and Webb et al.^8^ The literature however reports a lower average prevalence for stimulant use in non-medical undergraduate students.^5,8^ We hypothesise that students possibly used stimulants to increase academic performance, as was reflected in the reasons for stimulant use put forward by students; with very few using it for reasons other than to increase concentration. The extreme academic pressure experienced by the medical fraternity as well as high expectations to perform well might be possible reasons for the higher prevalence of stimulant use in medical students compared with non-medical students.^2^

We also found an increased prevalence of high-risk behaviour (concomitant use of illicit substances) in the group using stimulants. This finding mirrors results of other international reports on this topic.^5,6^ Although there were no statistically significant differences between the user and non-user groups pertaining to psychiatric or cardiovascular disease, it is disconcerting that there are medical students with heart and psychiatric disease using symphatomimetics without a script. This could be deleterious with reference to side effects and drug interactions.

In comparison to other reports,^5,6^ we did not find a higher use of stimulants in men, members of residential facilities, or students with lower grade averages. We found that more second-year than fourth-year students used stimulants. Stimulants were mostly obtained from ‘shops’ and ‘house shops’. It is concerning that some students reported obtaining stimulants from pharmacies without a prescription, as well as buying it online via Swiss Guard. It is also clear that many siblings are diverting stimulants to family members.

The authors found the differing opinions on stimulant use interesting. It might be expected that users and non-users would feel different on issues pertaining to non-medical stimulant use, but whether stimulant use per se influenced their viewpoint or vice versa remains to be explored.

Limitations of this study include the use of self-report questionnaires. Given that using stimulants without a valid script as well as selling scheduled medicine as part of diversion behaviour is illegal, the students might have been reluctant to answer honestly. Students may also have been reluctant to self-report a psychiatric diagnosis, hence the low number of participants reporting a diagnosis of ADHD. Although the results of this study may not be extrapolated to other universities or colleges, our results were comparable to other international reports.^2,8^

## Conclusion

Our study shows that the non-medical use of stimulants among medical students at this South African university is prevalent. This practice is not without risk however and carries ethical and policy-making implications. Duke University in North Carolina, USA, recently prohibited the non-medical use of prescription stimulants for academic purposes, classifying it as ‘cheating’.^11^ This raises the question whether South African universities and colleges should consider having a policy or protocol on the use of stimulants for cognitive enhancement. A policy such as this would likely be received with diverse responses, as the students in our study had differing opinions on what their university’s approach should be.

Future research should focus on assessing rates of stimulant use at other South African institutions and under non-medical students.
